# Family involvement on nursing wards and the role conflicts experienced by surgical nurses: A multicentre cross‐sectional study

**DOI:** 10.1111/scs.13032

**Published:** 2021-09-10

**Authors:** Marte A.A. Smits, Edwin J. Boezeman, Karen Nieuwenhuijsen, Angela G.E.M. de Boer, Els J.M. Nieveen van Dijkum, Anne M. Eskes

**Affiliations:** ^1^ Nursing Sciences Program in Clinical Health Sciences Utrecht Utrecht University Utrecht the Netherlands; ^2^ Department of Surgery Reinier de Graaf Hospital Delft the Netherlands; ^3^ Department of Surgery Cancer Center Amsterdam Amsterdam UMC, University of Amsterdam Amsterdam the Netherlands; ^4^ Department of Public and Occupational Health Coronel Institute of Occupational Health Amsterdam Public Health Research Institute Amsterdam UMC Location AMC Amsterdam the Netherlands; ^5^ Menzies Health Institute Queensland and School of Nursing and Midwifery Griffith University Gold Coast Australia

**Keywords:** cross‐sectional study, family participation, nurses, role problems, surgery

## Abstract

**Objective:**

To examine among surgical nurses whether work–role conflict, work–role ambiguity, respect, distress and trust in collaboration due to interactions with family caregivers in the nursing ward are associated with the quality of contact with patients and their families.

**Methods:**

A multicentre cross‐sectional study was conducted between January and March 2020. Surgical nurses completed a questionnaire recording work–role conflict, work–role ambiguity, sense of respect, distress, trust in collaboration and quality of contact with patients and their families. Data were analysed using correlation analysis, multiple linear regression analysis and mediation regression analysis.

**Results:**

A total of 135 nurses completed the questionnaire. The correlation analysis showed significant correlations between nurses’ impaired quality of contact with patients and their families and nurses’ work–role conflicts, work–role ambiguity, trust in collaboration and distress (*p* < 0.05). The multiple regression analyses corroborated that work–role conflict and distress were significantly and positively associated with impaired quality of contact. Furthermore, mediation regression analysis showed that work–role conflict was associated indirectly and significantly with quality of contact through distress.

**Conclusion:**

Work–role conflict due to having family caregivers involved in the care of hospitalised patients is significantly associated with nurses’ distress and quality of contact with patients and their families.

## INTRODUCTION

Worldwide, over 310 million surgical procedures are performed each year [1]. For patients, surgical procedures often involve a substantial loss of physical, emotional and social capacity [2]. Patients’ recoveries start immediately after surgery, but full recovery to their preoperative states of well‐being may take additional weeks or months after discharge from the hospital [3, 4]. After discharge, surgical patients are often cared for by family caregivers. However, family caregivers are known to also provide care during the hospitalisation period, despite often not feeling well trained nor fully self‐confident to do so [5, 6]. For instance, family caregivers provide emotional care to patients (such as comforting the patient when needed) and consult nurses on behalf of the patient about treatment options. Furthermore, surgical procedures may involve complications for patients; for instance, delirium, pneumonia, pressure ulcers and malnutrition [6, 7, 8]. Basic care delivered to hospitalised patients by their family caregivers, including help with personal hygiene, feeding, dressing and ambulating [7, 8], helps in preventing complications for patients after surgery [9]. Research shows that the involvement of family caregivers in the care of hospitalised patients yields positive patient outcomes, such as improved satisfaction and knowledge, reduced pain levels, less post‐operative complications and decreased stress and anxiety [10, 11, 12, 13].

The positive contributions of family caregivers to the welfare of patients are being acknowledged, and accordingly, family caregivers are encouraged to become involved in a patient's care during hospitalisation [10]. Nevertheless, potential negative consequences should not be overlooked [14]. Nurses, for instance, may experience work–role problems, such as work–role conflict, work–role ambiguity and distress, due to the involvement of family caregivers in the care of hospitalised patients. It is relevant to address this issue, for instance, because work–role conflicts may undermine the quality of caregiving of nurses. Should family involvement in the caretaking for hospitalised patients hinder nurses during their work, then it would become important to, for instance, develop guidelines for family involvement in the nursing ward and to offer nurses who interact with family caregivers relevant means and support at work.

‘Work–role conflict’ refers to having conflicting work responsibilities, while ‘work–role ambiguity’ refers to uncertainty about how to complete a work task [15, 16]. According to role theory [15], work–role conflict and work–role ambiguity are stressors that undermine the functioning of workers. Previous research conducted among nurses demonstrated that work–role conflict and work–role ambiguity decrease job satisfaction and increase emotional exhaustion, feelings of job‐related strain and distress [17, 18]. Distress in itself adversely affects work productivity, patient safety and quality of healthcare [19, 20]. For different reasons, nurses may experience work–role conflict and work–role ambiguity due to the involvement of family caregivers in the care of hospitalised patients. For instance, nurses who are assigned, or who assume, the responsibility of involving and guiding the family caregivers who are active in their nursing ward may feel that this is in conflict with the responsibility of having to provide nurse‐specific patient care [9]. Furthermore, nurses may experience ambiguity about how to cooperate with family caregivers during work or may fear the loss of authority due to the involvement of family caregivers in the care of hospitalised patients [21, 22, 23, 24, 25]. To date, it has not been examined among nurses whether role conflicts and role ambiguity due to interactions with family caregivers of hospitalised patients are associated with distress and impaired quality of contact with patients and their families.

Nevertheless, it is also important to address the ways in which family caregivers of hospitalised patients may contribute positively to the job attitudes and quality of caregiving of nurses. ‘Respect’ refers to the sense of being valued [26, 27], and workers react positively to respect received from others, because it instils feelings of self‐esteem and self‐worth [26, 27]. Studies conducted among workers, for instance, demonstrated that those who feel respected within their organisation reported job satisfaction and work motivation (e.g. intent to remain with the organisation) [27, 28]. Previous research has addressed the positive effects of respect received from members of the work team (e.g. the supervisor or coordinator), but respect may also be helpful in bringing group members of different types closer to each other [27]. Accordingly, it is of interest to examine among nurses whether respect received from family caregivers of hospitalised patients is associated with trust in the collaboration with family caregivers and the quality of caregiving.

In summary, the aim of this study was to examine whether negative (i.e. work–role conflict, work–role ambiguity and distress) and positive (i.e. feeling respected and trust in collaboration) factors in the involvement of family caregivers are associated with surgical nurses’ quality of caregiving (i.e. contact with patients and their families).

## METHODS

### Design and participants

Between January and March 2020, a multicentre cross‐sectional survey was conducted. Two Dutch academic medical centres, one teaching hospital and one peripheral hospital, allowed the researcher MAAS and the local research coordinators (HvdW, HvN and MdJ; see Acknowledgements) to recruit surgical nurses for research participation within these hospitals. The inclusion criteria for participants were (1) having a paid job as a surgical nurse on a surgical ward; (2) the ability to read and speak Dutch well; and (3) interactions with patients’ family caregivers during work activities. Furthermore, the nurses approached for research participation by MAAS had a qualification at level 4 or 6 on the European Qualifications Framework [29]. Power analysis conducted with GPower [30] showed that at least 84 participants would be required to provide complete data in order to achieve sufficient power (≥0.80) to detect medium effects with the statistical tests of the research (i.e. correlation analysis, multiple regression analysis involving three predictors).

### Data collection

Researcher MAAS and the local research coordinators (HvdW, HvN and MdJ) passed the printed research materials (i.e. invitation to participate, information letter about the research, informed consent form, questionnaire for completion after informed consent) to surgical nurses via their personal mailbox on the nursing ward. Furthermore, the nursing wards of the aforementioned hospitals were regularly visited or contacted to inform the surgical nurses about the research and to give the nurses the research materials after their having expressed willingness to participate. In addition, the invitation to participate in the research was distributed via email twice among the surgical nurses by the head nurses of the nursing wards, and this reminded the nurses about the research and encouraged participation. Hardcopy (printed) questionnaires were distributed, as it is known that the use of such questionnaires leads to a higher response rate than the use of online questionnaires [31, 32]. For the participants, the completion time of the questionnaire was approximately 15 min. After having completed the questionnaire, the participants submitted it to MAAS directly during her visit to the nursing wards or indirectly via the ‘return box’ that MAAS placed on the nursing wards.

### Instruments

The questionnaire included factual questions (e.g. what is your age?, what is your tenure?) for recording the sociodemographic and professional characteristics of the participants, and validated measurement instruments for assessing work–role conflict, work–role ambiguity, respect received from family caregivers, distress, trust in collaboration and nurses’ quality of contact with patients and their families. Where needed, scale headings and item wordings were adapted to make the measures appropriate for this research.

Nurses’ quality of contact with patients and their families (abbreviated to ‘quality of contact’) was measured with the 8‐item scale ‘Impaired contact with patients and their families’, (α = 0.81) of the Nurses Work Functioning Questionnaire (NWFQ) [33]. This instrument yields a single score and uses various 7‐point Likert scales (e.g. 1 = Never, 7 = Always) for recording the answers of participants to its various items [33]. A high score is suggestive of impaired quality of contact [33]. The word ‘family’ was added to three of its eight items (see Appendix 1).

Work–role conflict and work–role ambiguity were respectively measured with the relevant 3‐ and 4‐item scales of the Questionnaire on Organisational Stress‐D (VOS‐D) (work–role conflict: α = 0.69; work–role ambiguity: α = 0.66) [34]. The scales record the answers of the participants to their items on a 5‐point Likert scale (work–role conflict: 1 = Always, 5 = Never; work–role ambiguity: 1 = Very precise, 5 = Not at all) [34]. All the items of the work–role conflict scale were reverse scored. For each measure, a high score is suggestive of work–role problems (work–role conflict, work–role ambiguity). We added a specific answer heading to the measures, and in the items, the word ‘supervisor’ was changed to ‘family caregiver’ (see Appendix 1).

Respect received from family caregivers was measured with the relevant 4‐item instrument for recording ‘respect’; in previous research, this measure was found to have good internal consistency (0.86 ≤ α ≤ 0.97) [27]. The instrument records the answers of the participants to its items on a 7‐point Likert scale (1 = Totally disagree, 7 = Totally agree) [27], with a high score being suggestive of a sense of being respected. In the items, the word ‘coordinator’ was changed to ‘family caregiver’, and the word ‘volunteer’ was changed to ‘nurse’ (see Appendix 1).

Distress was measured using the 6‐item Stress–Energy Questionnaire, which has a calculated Person Separation Index (PSI, reliability coefficient) of 0.87 [35]. The answers of the participants to the items of the instrument were recorded on a 7‐point Likert scale (1 = Totally disagree, 7 = Totally agree) [35]. Items 4, 5 and 6 were reverse scored, with a high score suggesting distress. No item‐wording adjustments were made (see Appendix 1).

Trust in collaboration with family caregivers (abbreviated to ‘trust in collaboration’) was recorded with a measure adapted from the 4‐item Trust in Team Members measure, which was previously found to have good internal consistency (α > 0.70) [36]. The answers of the participants to the items of the measure were scored on a 7‐point Likert scale (1 = Totally disagree, 7 = Totally agree) [36]. A high score indicates trust in collaboration. In the items, the words ‘team member’ were changed to ‘family caregivers of patients’ (see Appendix 1).

### Analysis

#### Data handling

Data entry, recoding of reverse‐worded items and data analysis were performed with the IBM SPSS statistics software version 26 for Windows [37]. Reliability analyses were conducted to inspect the internal consistency of the measures. Subsequently, mean scale scores were calculated for use in data analysis. Missing data were handled with listwise deletion (complete case analysis).

#### Statistical analyses

First, Pearson correlation analysis was conducted, and an association matrix was construed. Correlation coefficients were considered to be small for values below 0.20, of medium size for values between 0.20 and 0.30 and large for values of 0.30 or higher [38]. Subsequently, after verification that assumptions were met, bootstrapped multiple regression analyses (bootstrap = 5000) were conducted to further examine correlations between the variables of the research. The first multiple regression analysis model (Model 1) was tested to examine whether work–role conflict, work–role ambiguity and respect received from family caregivers were related to impaired quality of contact between nurses and patients and their families, while they (i.e. role conflict, work–role ambiguity and respect) controlled for each other. The second multiple regression analysis model (Model 2) was tested to examine whether nurses’ distress and trust in collaboration were related to nurses’ quality of contact with patients and their families, while they (i.e. nurses’ distress and trust in collaboration) controlled for each other. In addition, as supplementary analysis, multiple regression models were tested to examine whether work–role conflict, work–role ambiguity and respect received from family caregivers were related to distress (Model 3) and trust in collaboration (Model 4), while they (i.e. work–role conflict, work–role ambiguity and respect) controlled for each other. Furthermore, there was a theoretical possibility of indirect correlations between variables and indirect correlations between variables. If variables correlate indirectly with each other, there is an intermediate variable linking the correlating variables, and this can be investigated with bootstrapped mediation regression analysis [39]. Accordingly, to examine indirect correlations, bootstrapped mediation regression was conducted with the relevant PROCESS macro for SPSS [39].

### Ethical approval

The Medical Ethics Committee of the Academic Medical Center judged (reference number: W19_477 #19.551) that a comprehensive evaluation was not required, as this study was not subject to the Medical Research Involving Human Subjects Act. The study was conducted in line with the principles of the Declaration of Helsinki (version 7, 2013) and in accordance with the Amsterdam UMC Research Code. Participants (nurses) provided informed consent by reading the information letter for the research and answering with ‘yes’ the statement ‘I give permission to use my data for this research included in the questionnaire. The data are processed anonymously’. Nurses who did not answer, or who answered ‘no’ to this statement, were not included in the research. The study is reported in line with the relevant STROBE criteria (STrengthening the Reporting of OBservational studies in Epidemiology) [40].

## RESULTS

### Participants

Of the 280 nurses who were handed a questionnaire, 135 returned it (response rate = 48%). Fifty‐two of these nurses (38%) worked in a teaching hospital, 44 (33%) in an academic hospital and 39 (29%) in a peripheral hospital. The data set included 119 female participants and 16 male participants, and the median age in the sample for the participants was 26 years (interquartile range [IQR] = 11). Eighty‐one nurses (60%) had experience with family caregiving in their personal life, and 70 (52%) stated that they had at least regular contact with family caregivers during work activities. Table 1 shows an overview of the characteristics of the research participants.

**TABLE 1:** Sociodemographic and professional characteristics

	Total (N = 135) Median (IQR)/*n* (%)
Age	
Missing *n* = 1	26 (11)
Gender	
Female	119 (88)
Hospital	
Academic	44 (33)
Teaching	52 (38)
Peripheral	39 (29)
Current function	
Senior nurse* (registered)	34 (25)
Nurse (registered)	79 (58)
Student	13 (10)
Other**	9 (7)
Contractual working hours per week	32 (4)
Work experience in total (in years)	4 (10)
Work experience on current ward (in years)	
Missing *n* = 2	2 (7)
Experience with family caregivers during work activities	
Rarely	16 (12)
Now and then	47 (35)
Regularly	38 (28)
Often	24 (18)
Very often	8 (6)
Missing *n* = 1	
Experience with a family caregiver programme	
Yes	25 (18.5)
No	108 (80)
Missing *n* = 2	
Experience with family caregivers in personal life	
Yes	81 (60)
No	52 (38.5)
Missing *n* = 2	

Abbreviation: IQR, interquartile range.

* Senior nurse is a general nurse with advanced tasks, for example to secure quality of care, to provide transcending care, to coach colleagues.

** Other functions were oncology nurses (*n* = 7), geriatric nurse (*n* = 1) and head nurse (*n* = 1).

### Internal consistency of the measurement instruments

We recalculated Cronbach's Alpha for all measurement instruments. Overall, the values of the reliability coefficients of the measures used in this research were in line with the relevant values of internal consistency observed in previous studies (see Appendix 2) [41].

### Correlation analysis

Table 2 shows the results of the correlation analysis. The correlation analysis showed initial significant (*p* < 0.05) correlations between the participants’ impaired quality of contact with patients and their families and the participants’ work–role conflict (*r* = 0.28), work–role ambiguity (*r* = 0.18), trust in collaboration (*r* = −0.18) and distress (*r* = 0.35). The correlation of distress with impaired quality of contact with patients and their families was strong. Furthermore, work–role conflict correlated moderately strongly with impaired quality of contact, while work–role ambiguity and trust in collaboration had only small correlations with impaired quality of contact. However, the correlation analysis showed no significant direct association between respect received from family caregivers of hospitalised patients and impaired quality of contact with patients and their families (*r* = 0.14, *p* = 0.12).

**TABLE 2:** Correlations

	M	SD	1	2	3	4	5	6
1. Work–role conflict	2.34	0.68	‐					
2. Work–role ambiguity	2.46	0.62	0.19*	‐				
3. Respect received from family caregivers	5.72	0.67	−0.16	−0.23**	‐			
4. Distress	3.29	0.99	0.32***	0.04	−0.17	‐		
5. Trust in collaboration with family caregivers	4.48	0.79	−0.26**	−0.05	0.29***	−0.16	‐	
6. Impaired contact with patients and their family	38.58	10.40	0.28**	0.18*	−0.14	0.35***	−0.18*	‐

* *p* ≤ 0.05.

** *p* ≤0.01.

*** *p* ≤0.001.

### Bootstrapped multiple regression analysis findings

Table 3 displays the results of the multiple regression analyses. The first model (*R* ^2^ = 0.10) showed that work–role conflict (β = 0.24, *p* < 0.01; 95% CI: 0.98–6.26) was associated significantly and directly with impaired quality of contact with patients and their families, but that work–role ambiguity (β = 0.14, *p* = 0.13; 95% CI: −0.67 to 5.31) and respect received from family caregivers (β = −0.08, *p* = 0.38; 95% CI: −3.89 to 1.48) were no longer associated significantly with impaired quality of contact with patients and their families (Table 2).

**TABLE 3:** Multiple linear regression analysis Results

Variable	*β*	95%‐CI Lower bound	95%‐CI Upper bound	*R*²
Model 1: DV = Quality of contact				0.10
Work–role conflict	0.24**	0.98	6.26	
Work–role ambiguity	0.14	−0.67	5.31	
Respect	−0.08	−0.39	1.48	
Model 2: DV = Quality of contact				0.14
Distress	0.38***	1.75	5.26	
Trust in collaboration	−0.12	−3.75	0.56	
Model 3: DV = Distress				0.12
Work–role conflict	0.31***	0.20	0.68	
Work–role ambiguity	−0.05	−0.35	0.20	
Respect	−0.15	−0.45	0.02	
Model 4: DV = Trust in collaboration				0.14
Work–role conflict	−0.25*	−0.44	−0.06	
Work–role ambiguity	0.04	−0.17	0.26	
Respect	0.28***	0.13	0.52	

* *p* ≤ 0.05.

** *p* ≤ 0.01.

*** *p* ≤ 0.001.

The second model (*R* ^2^ = 0.14) showed that distress (β = .38, *p* < 0.001; 95% CI: 1.75–5.26) was significantly and directly associated with the quality of contact with patients and their families, but also that trust in collaboration (β = −0.12, *p* = 0.15; 95% CI: −3.75 to 0.56) was no longer associated significantly with impaired quality of contact with patients and their families.

The third model (*R* ^2^ = 0.12) showed that work–role conflict (β = 0.31, *p* < 0.001; 95% CI: 0.20–0.68) was significantly and directly associated with distress, but that work–role ambiguity (β = −0.05, *p* = 0.59; 95% CI: −0.35 to 0.20) and respect received from family caregivers (β = −0.15, *p* = 0.09; 95% CI: −0.45 to 0.02) were not directly associated with distress when controlling for work–role conflict.

The final model (Model 4; *R* ^2^ = 0.14) showed that work–role conflict (β = −0.25, *p* < 0.05; 95% CI: −0.44 to −0.06) and respect received from family caregivers of hospitalised patients (β = 0.28, *p* = 0.001; 95% CI: 0.13–0.52) were significantly associated with trust in collaboration, while work–role ambiguity had no significant direct association with trust in collaboration (β = 0.04, *p* = 0.69; 95% CI: −0.17 to 0.26).

### Bootstrapped mediation regression analysis findings

**FIGURE 1:**
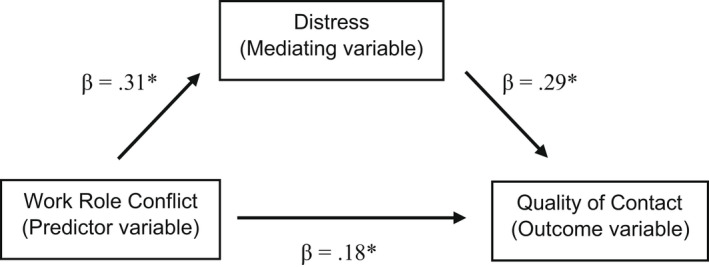
‘Mediation model’. Notes: *p < 0.05; The significant regression coefficient of the indirect effect of work–role conflict on quality of contact through distress is obtained by multiplying the relevant direct effect coefficients (0.31 *0.29 = 0.09)

In view of the results of the bootstrapped multiple regression analyses, it was considered relevant to test with bootstrapped mediation regression analysis only whether work–role conflict was related to impaired quality of contact with patients and their families via distress as a mediating variable, and such an association was identified (β = 0.09, *p* < 0.05, CI 95%: 0.04–0.16), see Figure 1. More specifically, while the bootstrapped mediation regression analysis showed work–role conflict (β = 0.18, *p* < 0.05) to have a direct association with impaired contact beyond distress (β = 0.29, *p* < 0.05) (*R* ^2^ = 0.15), the analysis also showed that work–role conflict and impaired quality of contact were associated indirectly with each other via (or due to) distress (β = 0.09, *p* < 0.05, CI 95%: 0.04–0.16).

## DISCUSSION

The aim of this study was to examine whether negative (i.e. role conflict, role ambiguity, distress) and positive (i.e. feeling respected, trust in collaboration) factors in the involvement of family caregivers in the care of hospitalised patients are associated with surgical nurses’ quality of caregiving (i.e. contact with patients and their families). The main finding of this research is that nurses who suffer work–role conflict and distress due to the involvement of family caregivers in the care of hospitalised patients may be more likely to report a decreased ability to maintain good contact with patients and their families.

### Contribution, avenues for new research and practical recommendations

This research contributes new insights about the quality of caregiving of nurses, yields new knowledge about family involvement in caregiving and offers practical recommendations relevant for preventing that the involvement of family caregivers in the caring for hospitalised patients may negatively influence the functioning of nurses at work.

Family involvement is becoming more widespread in the hospital environment and represents an emerging new field in the area of Patient‐ and Family‐Centred Care (PFCC). In PFCC, family members of patients are considered instrumental to the care of patients [10, 42]. Many studies have addressed the contributions of family caregivers to the health and recovery of patients after hospital discharge. The current research focussed on the presence of family caregivers in the caring for patients in the hospital and examined its consequences for the quality of caregiving of surgical nurses as an indicator of the functioning at work of these nurses. Qualitative studies have indicated that nurses appreciate and value the contributions of family caregivers in the care of hospitalised patients, but also that nurses have raised concerns about the involvement of family caregivers in this context [14, 43, 44]. The current research showed the distress of nurses due to interactions with family caregivers during work related to the quality of caregiving of the nurses (i.e., quality of contact with patients and their family). Future research may now examine the factors at work and/or the personal characteristics of nurses that may mitigate or buffer the effect of the type of distress on the nurses’ quality of caregiving, for instance the nurse job type (i.e. different types of nurses may react in different ways to involvement of family caregivers in the caring for hospitalised patients), social support from the supervisor and colleagues at work, work–role overload and/or the use of adaptive coping during work. Accordingly, the involvement of family caregivers in the care of hospitalised patients requires careful assessment, along with the notion of how issues and barriers that emerge due to involving family caregivers in the care of hospitalised patients can best be addressed. For instance, job descriptions that clarify what is expected from nurses in dealing with family caregivers, concrete policies regarding family involvement, supervisor acknowledgement and support, relevant training and social norms for nurses *and* family caregivers that foster communication and cooperation may help nurses in dealing with family caregivers of hospitalised patients and prevent nurses from experiencing work–role conflicts and distress.

The current research contributes to the understanding of nurses’ work–role problems, including work–role conflict and work–role ambiguity, which are considered to be stressors that undermine the functioning of nurses at work [17]. Previous research has demonstrated that general work–role conflict and ambiguity increase distress and decrease job satisfaction among nurses [45, 46, 47]. The current research complements and extends this previous work. This study examined work–role conflicts and work–role ambiguity resulting from interactions with family caregivers of hospitalised patients. The former was found to be primarily associated with distress and impaired contact with patients and their families, while the latter was found to have only an initial association with distress and quality of contact. These findings are new to the literature and warrant further conceptualisation and research before firm conclusions are drawn. Specifically, research on the role of moderating variables is required. For instance, nurses who hold a more positive attitude towards the presence of family caregivers in the hospital may be less inclined to report distress due to work–role conflicts resulting from their interactions with family caregivers. Furthermore, given the findings of the current research, it is worthwhile to further examine, and to develop interventions for preventing, nurses’ work–role problems and distress resulting from their interactions with family caregivers of hospitalised patients.

### Study limitations

It should be acknowledged that the study had its limitations. First, the nonresponse in this research may represent a limitation of the study. Nonresponse undermines external validity in terms of the possibility that the nonresponding individuals have specific shared features that do not apply to those who completed the questionnaire. At the same time, it should be acknowledged that the response rate of the current research is in line with response rates previously found in studies conducted among surgical nurses [48, 49], and therefore, the findings of the current research still have value. Because the research participants were all Northern European surgical nurses working in a Western hospital setting, cross‐cultural research is needed to examine whether the findings and conclusions of this research also apply to nurses working in other work contexts and cultures. While general work–role conflicts have been found to evoke stress among workers living in a collectivistic or family‐oriented culture [50], it may be the case that in such cultures, nurses are more understanding of family involvement and therefore less inclined to suffer work–role conflicts and distress due to family involvement in the care of hospitalised patients. In the current study, the internal consistency of the measure recording the nurses’ quality of contact with patients and their family had a moderate Cronbach's alpha value of between 0.60 and 0.70 [51], while an exploratory factor analysis made clear that the measure that recorded the nurses’ trust in collaboration with family caregivers was still unidimensional. Accordingly, the use of these measures was considered acceptable in the current research and has helped in obtaining insights new to the literature. Another limitation of the research is that only self‐reported quality of contact with patients and their families was recorded as an indicator of nurses’ quality of caregiving, with no additional indicators nor objective indicators of the quality of caregiving of nurses (such as supervisor‐rated performance scores). Furthermore, the current research should be considered to be a first step and first insight into this new area, and its findings may encourage others to start more extensive studies. Finally, this study was a cross‐sectional study, and accordingly, no causal effects were examined.

## CONCLUSION

It can be concluded that the involvement of family caregivers in the care of hospitalised patients plays a role in how nurses both experience and function in their jobs. Specifically, work–role conflicts due to interactions with family caregivers of hospitalised patients were found to be associated with nurses’ distress and impaired quality of care. This should be taken into consideration, and addressed, when involving family caregivers in the care of patients in hospital. Nurses can be trained regarding interactions with family caregivers, and supervisor acknowledgement and support should be given with regards to the changing roles that nurses experience when family caregivers are available.

## CONFLICT OF INTEREST

The authors declare no conflicts of interest.

## AUTHOR CONTRIBUTION

1. All authors are involved in conception or design of the work, critical revision of the article and final approval of the version to be published.
2. SMITS performed the data collection.
3. SMITS, BOEZEMAN and ESKES involved in data analysis and interpretation and drafted the article.

## APPENDIX 1 Relevant background information and adjustments per scale

**Table:**

Variable	Scale	Year	Authors	Original validated population	Cronbach's Alpha or PSI	Adjustments of scales
Nurses’ quality of contact with patients and their family	‘Impaired contact with patients and their family’ from NWFQ [31]	2011	Gärtner, Niewenhuijsen, van Dijk and Sluiter	Nurses	α = 0.81	The term ‘*family’* was added in three of the eight items to make the scale more relevant to the specific context of this study. For example ‘*family’* was added in the phrase: I do not succeed in listening well to my patients and family.
Work–role ambiguity	Vragenlijst Organisatie Stress‐Doetinchem: VOS‐D [32]	1986	Bergers, Marcellissen and de Wolff	Nurses	α = 0.66	We added a specific answer heading, for example *‘Concerning family caregivers during work activities*,*…*’. For example: ‘Concerning family caregivers during work activities, do you ever receive conflicting assignments?’ Another question in the original scale was formulated as followed: ‘Do you know exactly how your *supervisor* feels about your performance?’ The term ‘*supervisor’* was replaced with ‘*family caregiver’*.
Work–role conflict	Vragenlijst Organisatie Stress‐Doetinchem: VOS‐D [32]	1986	Bergers, Marcellissen and de Wolff	Nurses	α = 0.69	
Respect received from family caregivers	Respect [26]	2014	Boezeman and Ellemers	Volunteer workers	0.86 ≤ α ≤ 0.97	To record whether nurses feel respected by the family caregivers of patients, we replaced in the item‐wording the term ‘*coordinator’* with ‘*family caregiver’* and the term ‘*as a volunteer*’ to ‘*as a nurse’*. For example: ‘My *coordinator* values my contribution *as a volunteer’* into ‘The *family caregiver* values my contribution *as a nurse’*.
Distress	Stress‐Energy Questionnaire [33]	2005	Hadzibajramovic, Ahlborg jr, Grimby‐Ekman and Lundgren‐Nilsson	Public healthcare organisation and social insurance office workers	PSI = 0.87	No adaptations were made.
Trust in collaboration	Trust in team members [34]	2005	Schippers, den Hartog and Koopman	Team members	α > 0.70	We replaced the term ‘*team member*’ with ‘*family caregivers of patients’* in the item wordings. Nurses and family caregivers of patients are not formal team members of each other; hence, the adaptation is necessary to make the scale relevant to the context of this research.

## APPENDIX 2 Cronbach Alpha's* per scale

**Table:**

Variable	Scale	Original Cronbach Alpha in scale	Cronbach Alpha after minor adjustments
Quality of contact	‘Impaired contact with patients and their family’ from NWFQ [31]	α = 0.81	α = 0.69
Work–role Ambiguity	VOS‐D: Questionnaire on Organisational Stress‐D [32]	α = 0.66	α = 0.74
Work–role Conflict	VOS‐D: Questionnaire on Organisational Stress‐D [32]	α = 0.69	α = 0.69
Respect	Respect [26]	0.86 ≤ α ≤ 0.97	α = 0.92
Trust in collaboration	Trust in team members [34]	α > 0.70	α = 0.58
Distress	Stress‐Energy Questionnaire [33]	0.87 (PSI)	α = 0.87

Abbreviation: PSI, Person Separation Index.

* Cronbach's alpha (α) is a measure used to assess the reliability, or internal consistency, of a set of scale or test items.
